# Synchrotron XANES and EXAFS evidences for Cr^+6^ and V^+5^ reduction within the oil shale ashes through mixing with natural additives and hydration process

**DOI:** 10.1016/j.heliyon.2021.e06769

**Published:** 2021-04-14

**Authors:** Tayel El-Hasan, Messaoud Harfouche, Allayth Aldrabee, Nafeth Abdelhadi, Nizar Abu-Jaber, Giuliana Aquilanti

**Affiliations:** a Department of Chemistry, Mutah University, 61710, Mutah, Jordan; b SESAME Synchrotron, Allan, Al-Balq'a, P.O. Box 7, 19252, Jordan; c Jordan Atomic Energy Commission, Shafa Badran, Amman, Jordan; d Faculty of Engineering and Technology, Al-Balqa Technical University, Amman, Jordan; e Dept. of Civil and Environmental Engineering, German Jordanian University, Naour, Jordan; f Elettra - Sincrotrone Trieste, Area Science Park, 34149 Basovizza, Trieste, Italy

**Keywords:** XAFS, XANES, EXAFS, Oil shale ash, Additives, Hydration process, Cr(IV) reduction

## Abstract

Solid friable residues (i.e. Ash) from combusted oil shale are a major environmental issue because they are highly enriched with toxic elements following combustion. The synchrotron based techniques X-ray Absorption Fine Structure (XAFS) were used for determining the changes in speciation of Chromium (Cr) and Vanadium (V) in the ash and its mixtures with Red soil and Phosphogypsum as additives, through one-year period of hydration process. The X-ray Absorption Near Edge Structure (XANES) qualitative results indicate that all mixtures exhibits similar patterns showing that Vanadium has remain as pentavalent state, on the contrary Chromium has dramatic decreased from hexavalent to trivalent. This change in Cr speciation became clearer with increasing hydration period. Therefore, the results confirmed the advantage of the hydration process in the Cr(VI) reduction which might be due the domination of carbonate phase within all mixtures, thus hydration caused carbonate dissolution that increase the pH toward more alkaline which caused the Cr(IV) reduction into less-harmful and less mobile Cr(III). This increase in pH was not in favor of changing the V(V) into V(IV) due to its large stability field V(V). The Extend X-ray Absorption Fine Structure (EXAFS) analysis showed that Cr exhibiting a coordination shell of C-atoms as first nearest neighbors backscattering atoms around Cr, and at C-atoms backscattering at medium range order. This confirmed the domination of carbonate media through the best fitting of Cr–C. Which might be attributed to the more alkaline conditions developed during saturation of water (hydration), that accelerates of the reduction of Cr(VI) into Cr(III). This means simply that hydration of the ash can reduce the presence of harmful Cr(VI) in these ash tailings.

## 1 Introduction

Organic-rich black shales (i. e. Oil Shale) underline vast areas in Jordan; they are distributed in the north, central and southern parts of the country. El-Lajjoun is the main area containing the Oil Shale, its 110 km to the south of the capital Amman. Many described these deposits (e.g. Futyan, 1968; Weirsna, 1970; Bender, 1974; and Yassini, 1980, Abed and Ameirah, 1983; El-Hasan, 2008, El-Hasan et al., 2015).

**Figure 1:**
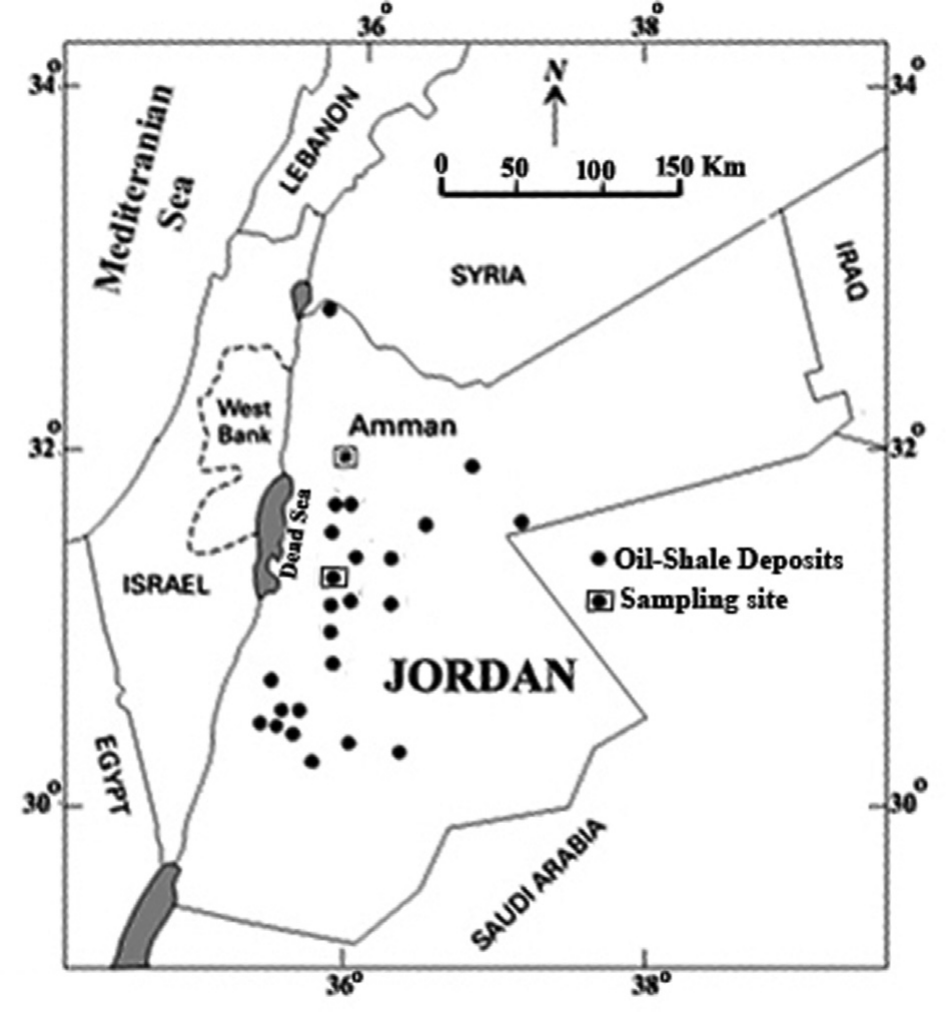
Location map showing the Oil shale deposits in Jordan, and the sampling site (*Modified by*Hamarneh, 2006).

Recently, Jordan country has started to use the Oil Shale for generating electrical energy through direct combustion, after surface mining of Oil shale at Attart Umm Al-Ghudran area Figure 1. Such power plant will provide Jordan with significant electrical power from a domestic source of fuel for the first time. No additional fuel is necessary due to the high caloric heat value and carbon burn-out as high as 99 %. A rough calculation suggests, however, that a 900 MW thermal power plant would require 6000 tons of oil shale. This would generate 3300 tons of burned oil shale (BOS) ash per day (i.e. 1.2 million tons per year). Oil shale utilization and the accumulated BOS ash may have a severe environmental impact, as it is friable and enriched in trace and toxic elements (El-Hasan, 2008, and El-Hasan et al., 2015). The fact that black shales are have potential economic metal accumulations and very important hosts for PGE (Pašava et al., 1993). Pasava (1990) reported higher trace elements concentrations in the Barrandian Upper Proterozoic (Bohemian Massif) accompanied with high organic carbon. Rimmer (2004) has reported enrichment of Mo, Pb, Zn, V, Cu, Cr, and Co within black shales in the central Appalachian Basin. Black shales serve as host for sulfur and other metal either through syngenetic or epigenetic mineralization. Coveney et al. (1992) concluded that the organic matter influences trace element enrichment in black shales. Many trace elements in black shales were used as indicators for anoxia especially Mo, V, U and Zn Dean et al. (1997). Alego and Maynard (2004) found that trace elements commonly exhibit considerable enrichment in laminated, organic-rich facies, especially those deposited under reduced sulfur rich conditions (i.e. euxinic conditions).

The BOS huge friable ash, tailings through the ultimate interaction with rain and groundwater, would form leachate that might resemble toxic mine drainage. This leachate might reach the country scarce surface and groundwater bodies causing harmful pollution. Besides, the large Oil shale industry would substantially aggravate the water supply problem in Jordan as it consumes large amount of water. Therefore, it is essential to analyze the mobility of toxic elements within BOS ash, in particular the chromium, which can be in the form of toxic chromate Cr (VI). Many previous studies have shown successfully the use of XANES for the discrimination of different oxidation states in chromium in treated wood (Strub et al., 2008), in addition to glass samples (Görner et al., 2006). This BOS ash was found to be getting more enrichment of trace and toxic elements due to the aerobic combustion process (El-Hasan, 2018). The chromium specious in the BOS ash was identified by synchrotron XANES to be Cr(IV), which represents the most hazardous pollution source (El-Hasan et al., 2011). US Environmental Protection Agency had considered Cr(VI) compounds as group A inhalation carcinogen (Chen et al., 2012). Wu et al. (2020) reported Cr(VI) as very contaminant because of its high solubility in water and mobility. Verbinnen et al. (2013) classified C(VI) among the most important oxidate in the CERCLA 2011 Priority List of Hazardous Substances.

Therefore, many researchers work extensively in order to encapsulate and immobilize the toxic elements particularly the Cr, the recent approach was performed using natural additives and hydration process (El-Hasan et al., 2019). However, the elimination of the harmful Cr(IV) could be done by adsorption, remediation or even reduction to Cr(III). Ibrahim et al. (2012) found that the bacteria Bacillus sp. KSUCr9a showed the ability of repeated bioreduction of chromate without any addition of exogenous nutrients, indicating its possible application in chromate detoxification. Chiou-Pin et al. (2012) Studied the effect of liming on the reduction of Cr(IV) in the agricultural soils, they found it insignificant factor. Akhtar et al. (2020) found that the most effective and cost-friendly method to remediate heavy metals Cr and Zn with a removal efficiency up to ~ 67% for Cr and ~ 55% for Zn at the end of 48 h is by using biologically prepared nano-composite of CuI which is a combination of both chemical and biological methods.

This work aimed to elucidate the effect of hydration and ageing process on Cr(VI) reduction through ageing period extended to 12 months. As will to investigate the reliability of using the synchrotron XAFS techniques to investigate the effect of hydration process on the Cr(IV) and V(V) reduction within the various BOS ash mixtures through one year aging periods.

## 2 Methodology and materials

### 2.1 Additives and mixtures

Burning of ambient Oil Shale quantity under aerobic combustion process up to 850 °C and 1000 °C and preparing the other additives (i.e. Red soil and Phosphogypsum). The Oil Shale samples were collected from El-Lajjoun area. The mixtures were prepared as follows:

○ BOS Ash alone hereafter termed as (ASH).
○ BOS Ash + Red Soil; 3:1 ratio hereafter termed as (ARS).
○ BOS Ash + Phosphogypsum; 3:1 ratio hereafter termed as (APG).

The Red soil was brought from Mutah University campus; meanwhile, Phosphogypsum was collected from Aqaba area, as it is piled up as huge tailings in Aqaba as a byproduct from the phosphoric acid manufacturing plant. These additives were used because they are natural, abundant, accessible and with low costs. These mixtures of the BOS ash, Red soil and phosphogypsum were prepared in 10 L plastic basins with ratios of 1:3 X:BOS ash (X stand for Red soil or Phosphogypsum). They kept in water saturation for 1, 3, 6 and 12 month period.

### 2.2 Additives preparations and analytical methods

#### 2.2.1 Ashing of oil shale (BOS production)

Performing the ashing process for the oil shale to produce the BOS under aerobic combustion process is the prime point to demonstrate the actual industrial production. This work package was done by collecting about 200 kg of the Oil shale samples from the El-Lajjoun area (Central Jordan). The Oil shale samples crushed, grinded, homogenized, and sieved to less than 150 μm. Then this powder fraction burned up to1000 °C for two hours using a muffle furnace. After the ashing process, the BOS samples were allowed to cool down in the desiccators under silica gel for 24 h.

#### 2.2.2 The ageing experiment (hydration)

Performing the ageing (hydration) experiments for the mixtures of BOS and natural additives samples of up to one-year duration carried out in open-air containers. Where a series of subsamples keptin fully water-saturated. The samples were periodically mixed to prevent the artifacts caused by slow diffusion effects upon solidification of the pastes. Sub-samples were taken at intervals of 1, 3, 6 and 12 months of hydration reaction. To prevent further hydration and mineral reaction, the samples dried in vacuum desiccators and stored under silica gel prior to measurement.

#### 2.2.3 Synchrotron measurements

The three used mixtures for all ageing periods were grinded and pressed into 11 mm diameter pellets. Both original BOS and aged mixture samples were analyzed for their Cr and V oxidation states using sed XANES technique. Furthermore, for determining the nature of Cr and V hosting materials matrix, Cr-rich containing mixture was investigated using the EXAFS technique.

XAFS measurements at the Cr- and V– K edges (5.989 and 5.465 keV respectively), for both the fresh BOS and all aged mixtures samples, were performed at XAFS beamline at ELETTRA – Italian synchrotron light facility (Di Cicco et al., 2009). Additional analysis were performed on the XRF/XRF beamline at Synchrotron-light for Experimental Science and Applications in the Middle East (SESAME), Allan, Jordan. The XAFS/XRF beamline is optimized for hard X-rays covering an energy range between 4.7 keV to 30 keV and dedicated for X-ray spectroscopic studies in all fields (Harfouche et al., 2018).

At both beamlines (XAFS, Elettra and XAFS/XRF, SESAME), XAFS data were collected in fluorescence mode using a single element silicon drift detector (SDD) from Ketek. A double crystal monochromator equipped with Si(111) crystal was used to tune the energy with a step size of 0.2 eV at the XANES region and variable steps to collect high quality EXAFS data up to k = ~13 Å^−1^. A minimum of three scans were taken for each sample to enhance the signal/noise ratio. Background subtraction and normalization of the XAFS data were performed by the ATHENA part of the DEMETER software package (Di Cicco et al., 2009). The WinXAS software package used for further modelling and structural fitting of the EXAFS data (Ressler, 1998). Reference samples were used for the redox comparison such as compounds with Cr(0), Cr(III), Cr(IV), V(IV), V(IV) species.

## 3 Results

### 3.1 Cr(VI) reduction into Cr(III)

**Figure 2:**
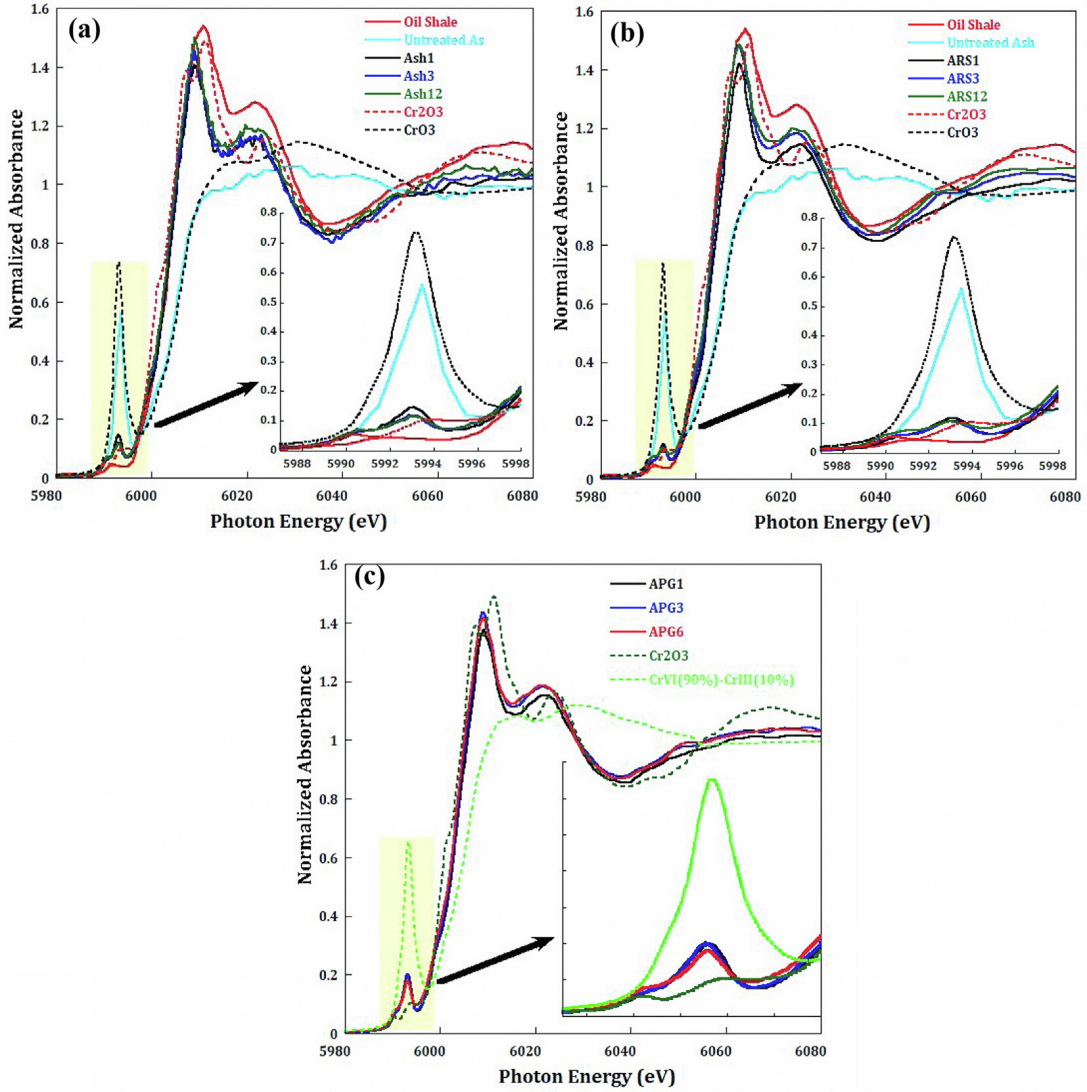
Normalized XANES spectra collected at the Cr– K edge exhibiting a Pre-edge peak (5993 eV) a white line peak (6010 eV) a post-edge peak (6025 eV) and partially the first EXAFS oscillation peak (~6070 eV) for a) ASH mixture of all ageing periods, b) ARS mixture of all ageing periods, and c) APG mixture of all ageing periods.

**Figure 3:**
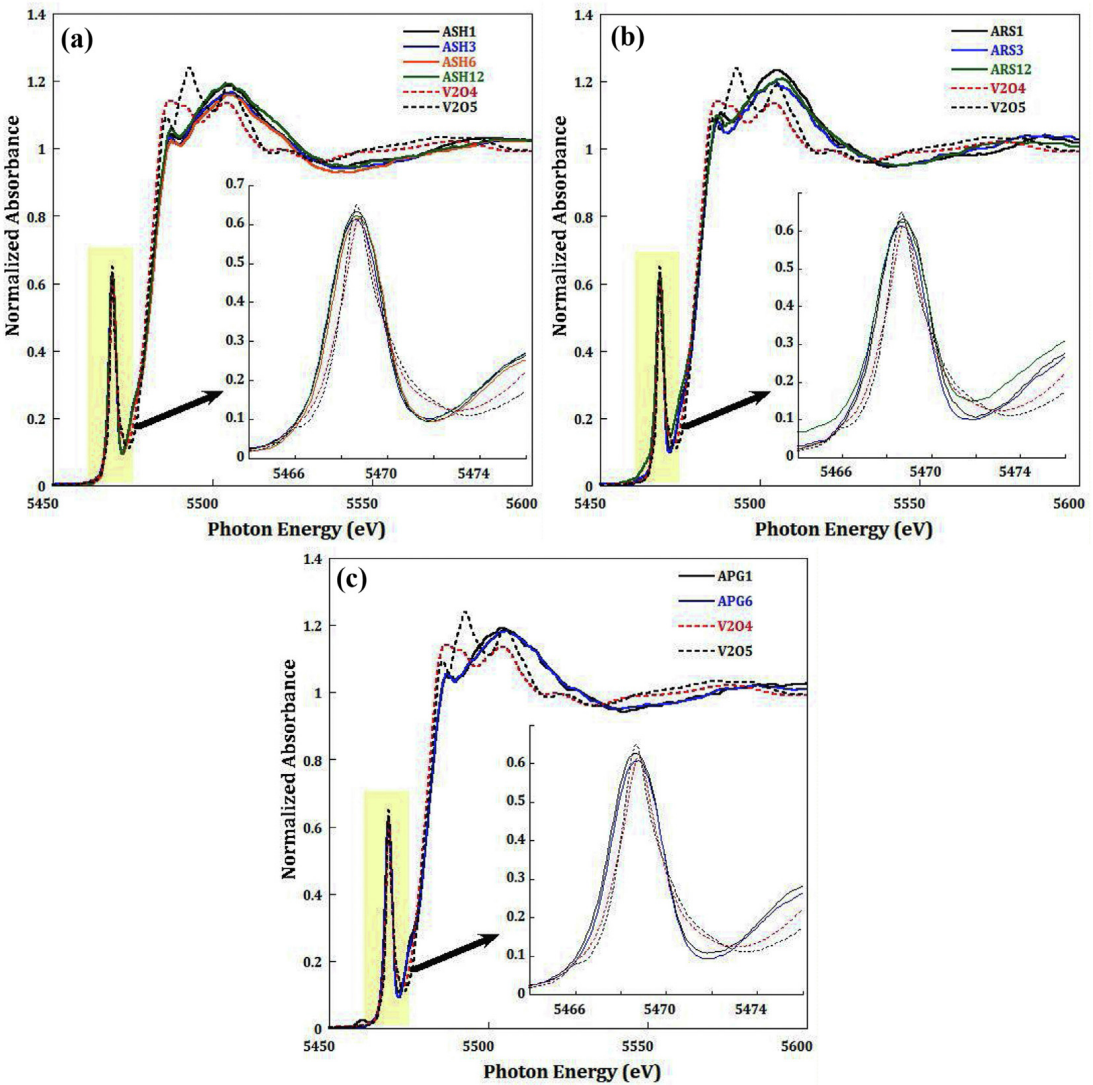
Normalized XANES data collected at V– K edge with zoom on pre-edge feature for a) ASH mixture of all ageing periods, b) ARS mixture of all ageing periods, and c) APG mixture of all ageing periods compared to reference samples. *Note: References XENES data were collected in different beamline as the samples where the resolution is slightly different.*

The XAFS is a powerful element-selective technique that is sensitive to the three-dimensional structure around the photo-absorber, and can effectively probe the defects, vacancies, and distortion in local structure. The experimental V and Cr K-edge XANES spectra of the fresh and BOS samples are marked mainly by three major peaks called pre-edge, white line and post edge at respective energies of 5993, 6010 and 6025 eV for Cr spectra and similarly at 5469, 5488 and 5506 eV for V spectra. In addition to a broad peak at higher energy called first EXAFS oscillation (Figures 2 and 3). Therefore, based on the pre-edge peak of the collected XANES spectra show that Cr found in all mixtures was of Cr(III) as shown in Figure (2a, b, and c), meanwhile the untreated ash sample is showing to contain more Cr(VI). It is worth to underline that the effect of leaching for all mixtures and there leached counterparts, exhibit similar pattern and intensity of Cr(III) peak resulting into a redox state ratio similar in all mixtures, as shown in Figure (2a, b, and c).

### 3.2 V(V) reduction to V(IV)

XANES data collected at the V K-edge show almost all ASH, ARS and APG mixtures are identical as shown in Figure (3a, b, and c). The pre-edge feature due to transition s → d at 5469 eV used as footprint for understanding changes in the redox state of atoms show no changes in all mixtures along the duration of hydration process, Vanadium remains in the pentavalent oxidation state V(V) which means no effect of hydration on V reduction.

### 3.3 Cr EXAFS results

As the investigated sample (ASH3) totally formed from BOS and thus dominated by carbonate materials before hydration (El-Hasan et al. 2011, 2015). This may indicate the phase in which Cr was embedded or hosted in (i.e. Calcite, Portlandite or Ettingrite). El-Hasan et al. (2019) has showed mineralogically and petrographically the presence of such mineral phases in these mixtures and they developed during hydration process with ageing. The results from EXAFS measurements for Cr are presented in Table 1.

**Table 1:** Cr-EXAFS Fourier Transform fitting results for a selected sample (ASH3).

Ligand	N (atom)	R (Å)	σ^2^ (Å^2^)	ΔE (eV)
Cr–C	6.0	2.03	0.0002	4
Cr–Cr	0.7	2.72	0.003	
Cr–Cr	4.1	3.70	0.008	
Cr–C	4.2	3.90	0.008	

**Figure 4:**
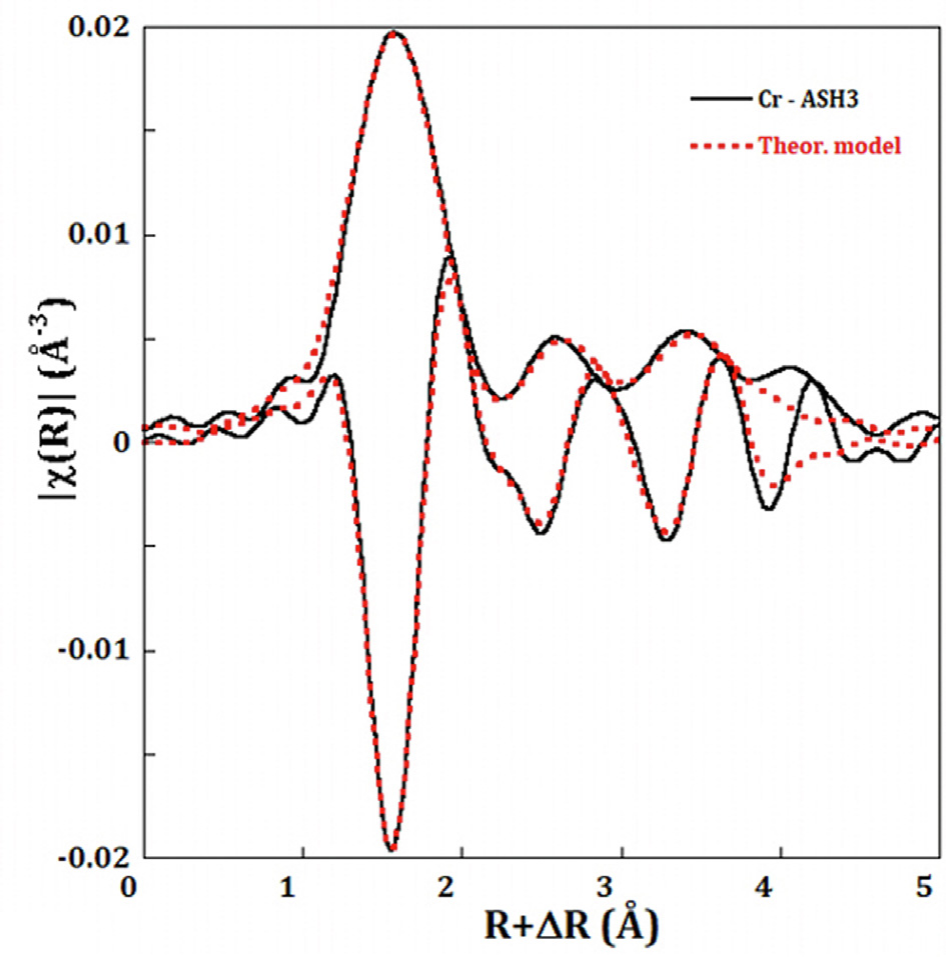
Fourier Transform (Magnitude and real part) of the EXAFS signal showing the best fited model to data.

For better understand the local structure around Cr atoms, rich Cr containing samples were investigated by EXAFS. The results of data fitting to the best structural model in (Figure 4) shows a coordination shell around Cr formed of 6 C-atoms at an interatomic distance Cr–C of 2.03 Å, followed by two shells of Cr backscattering next neighbors at distances of 2.72 Å and 3.7 Å. Furthermore, a probable signal from the C-atoms at longer distances (3.9 Å) could be also observed. These findings suggest a Cr–C structural form, which might fulfilled through the carbonate rich matrix of studied mixtures.

## 4 Discussion

### 4.1 The effect of dominated mineral phases of studied ashes

The XANES and EXAFS had clearly determined the speciation of Cr and V in the three studied mixtures. The carbonaceous nature of the oil shale ash was evident through previous work evident from XRD and SEM investigation. These results showed that besides calcite there is formation of complex cement-like mineral assemblages (e.g. Portlandite (Ca(OH)_2_) and Ettringite (Ca6Al2(SO4)3(OH) 12·26H2O) and other Calcium–Aluminum-Silica-Hydrated (C-A-S-H) compounds) (El-Hasan et al., 2019). Which indicates the domination of calcite that was approved by the EXAFS undulations that showed the presence of Cr–C bonds as the best fitted as shown in Figure 4. Therefore, calcite is the ultimate stable phase in this ash system.

Such mineral assemblages are capable to contained Cr and V within their mineral structures as approved from the lower leachability of Cr and V from these three mixtures, particularly ARS, which have the lower leachability rate (El-Hasan et al., 2019). Similarly (Yao et al., 2014), have reported the formation of calcium chromium mineral phase (e.g. CaCrO_4_) in various industries such as metallurgical, refractory, chemical and cement industries. Moreover (Mao et al., 2016), reported that CaCrO_4_ phase can be produced by promoting Cr(III) oxidation in the fly ash and reaction with CaO. Leisinger et al. (2010) reported that solid solution occurred through which CrO_4_^−2^ is substituting SO_4_^−2^ in the Ettringite.

### 4.2 The effect of hydration process (i.e. pH-Eh changes)

Over the pH of natural groundwater Cr(III) tend to be adsorbed on the surface complex (Oxyhydroxide). Therefore, hazard tends to be localized. But Cr(VI) is more stable as anionic species if no reductant is available. Thus, it will be more mobile and potentially more bioavailable (Peterson et al., 1997). Cr(VI) toxicity can be moderated through the reduction into Cr(III) (Deiana et al., 2007). In addition, because Cr(VI) has lower redox potential values at higher pH, then increasing pH is detrimental act to the reduction of Cr(VI) (Park et al., 2004; Su and Ludwig 2005). Therefore, more alkaline conditions can act as reductant of Cr(VI) and accelerate its reduction to Cr(III). This was obvious through ageing period ofthe three mixtures, as more Cr(III) pre-edge beak was found to increase with increase of ageing Figure (3a, b and c). Nevertheless (Ibrahim et al., 2012), was able to reduce Cr(VI) in a wide pH range (7–11) with an optimum growth and reduction yield at pH 9. Therefore, as we started with normal tap water in the hydration process then pH was measured as 7–7.5, which resembles that mentioned in (So et al., 2017) for the fresh river and lake water pH and Eh limitation.

**Figure 5:**
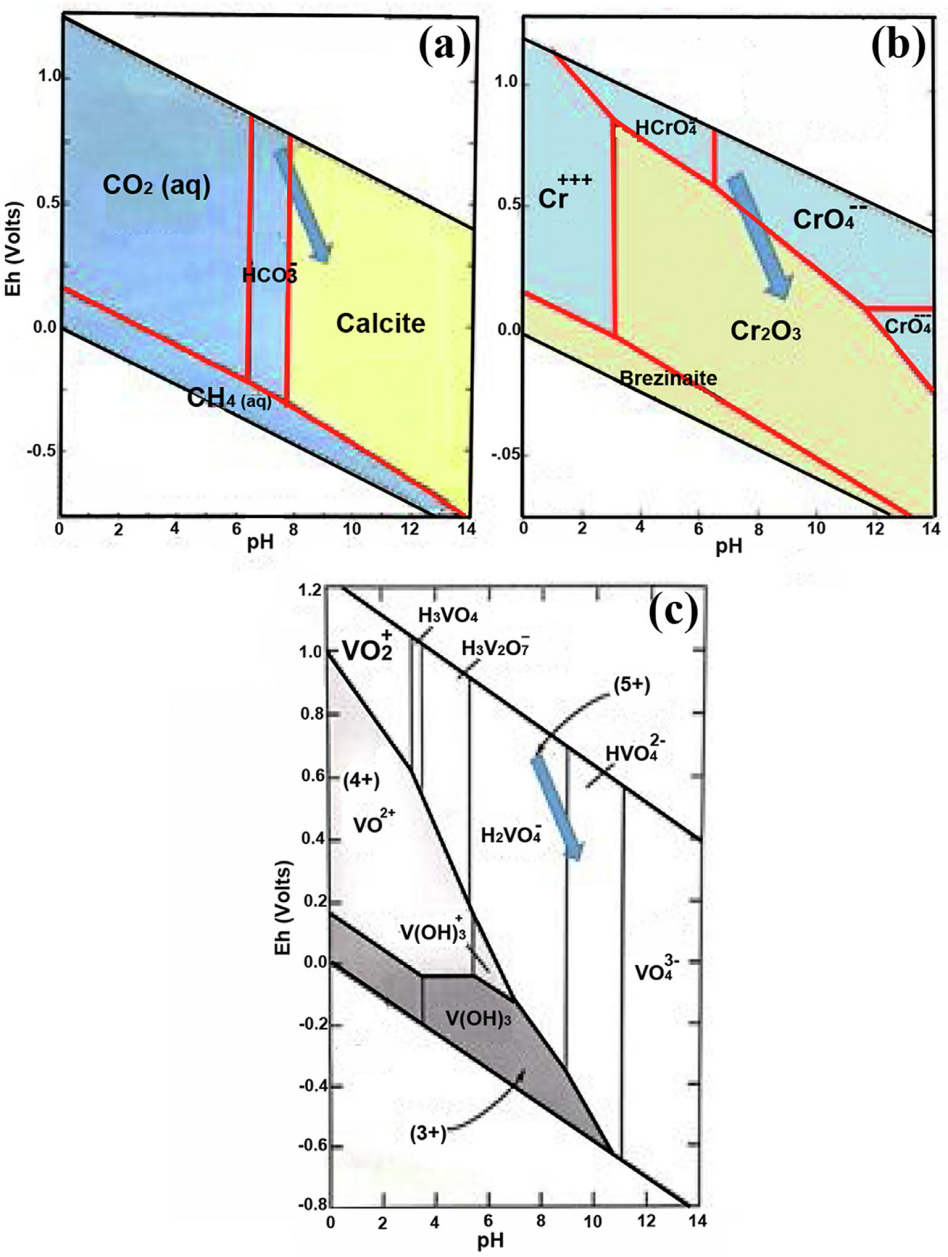
Predominance Eh-pH phase diagram for a) Calcite–CO_2_ system, b) Chromium species, and c) Vanadium species. (*Modified after*Brookings, 1988).

Experimentally, at equilibrium; water composition after dissolution of calcite in either closed system (H_2_O + Calcite) or in an open CO_2_ system (H_2_O + CO_2_+Calcite) at 25 °C, starts with neutral pH in the two systems results in increase of pH = 8.21 and pH = 9.91 for open and close system respectively (https://www.aqion.de/site/164). Kamei et al. (2010) in their interpretation of the natural analogues of cement in central Jordan found that interaction between groundwater with pH 7–7.5 and cement mineral assemblage causes formation of hyper alkaline solution pH = 12.5. Because the dissolution of CaCO_3_ with water dissociates to Ca^2+^ and CO_3_^2-^ the later would form solution that is more alkaline. Interestingly, the increase in pH are negatively correlated with Eh, similar notice was mentioned in (Van Breemen, 1987; Bohrerova et al., 2004). Therefore, the Eh-pH environment of the ash system during the hydration process where calcite tends to dissolute. Thus became more alkaline and reducing as it changed from mildly basic oxidizing environment towards more basic reducing environment Figure (a), like those mentioned in (Kamei et al., 2010; So et al., 2017). This might be attributed to that the increasing in pH was controlling factor causing the reduction of Cr(VI) to Cr(III) Figure 5b.

However, this was not valid for V(V) as the range of change in the Eh-pH stability does not cause reduction of its oxidation, due to its wide stability field as V(V) with regard to the range of change in the pH and Eh, thus it remains as pentavalent species V(V), Figure (5c). Mao et al. (2016) reported that the presence of alkaline earth metals beside SiO_2_ can accelerate the Cr(VI) reduction because of increase in pH; this is in agreement with our ash system which resembles that of cement (i.e. alkaline earth, Si–Al-hydrate and iron).

## 5 Conclusions

The synchrotron XAFS analytical results confirms the effect of increasing pH (i.e. Alkalinity) as controlling factor in Cr(VI) reduction process. EXAFS analysis provides evidences that Cr have been captured inside carbonate minerals formed due to hydration process mainly dominated by calcite, ettringite and portlandite. The XANES results, for all hydration ageing periods showed that hydration process with all ash mixtures had developed more alkaline environment accompanied with deceasing in Eh, subsequently caused the reduction of Cr(VI) to Cr(III), which means reducing the risk of Cr toxicity and mobility. This process can be described as a self-healing process through water addition to ash tailings, because hydration would dissolute the calcite in the ash and form cement-like minerals that would increase the alkalinity which is usually accompanied with lowering Eh values, thus facilitating the reduction of Cr(IV) to Cr(III) with time. However, the increase of alkalinity has no effect on V reduction, because of the wide stability pH and Eh field of V(V).

## Declarations

### Author contribution statement

Tayel El-Hasan: Conceived and designed the experiments; Analyzed and interpreted the data; Wrote the paper.

Messaoud Harfouche: Conceived and designed the experiments; Performed the experiments; Contributed reagents, materials, analysis tools or data; Wrote the paper.

Allayth Aldrabee and Giuliana Aquilanti: Performed the experiments; Contributed reagents, materials, analysis tools or data.

Nafeth Abdelhadi and Nizar Abu-Jaber: Analyzed and interpreted the data; Wrote the paper.

### Funding statement

This work was supported by the 10.13039/501100002385Ministry of Higher Education (SRF) - Jordan (BAS/01/1/2015), 10.13039/100014806Elettra-Sincrotrone Trieste - Italy (20170142) and SESAME Synchrotron Center - Jordan (20160025).

### Data availability statement

Data will be made available on request.

### Declaration of interests statement

The authors declare no conflict of interest.

### Additional information

No additional information is available for this paper.
